# Liposome-Encapsulated Bacillus Calmette–Guérin Cell Wall Skeleton Enhances Antitumor Efficiency for Bladder Cancer In Vitro and In Vivo via Induction of AMP-Activated Protein Kinase

**DOI:** 10.3390/cancers12123679

**Published:** 2020-12-08

**Authors:** Young Mi Whang, Da Hyeon Yoon, Gwang Yong Hwang, Hoyub Yoon, Serk In Park, Young Wook Choi, In Ho Chang

**Affiliations:** 1 Department of Internal Medicine, Seoul National University Hospital, Seoul 03080, Korea; 2 Department of Biochemistry and Molecular Biology, Korea University College of Medicine, Seoul 02841, Korea; dbsek1014@naver.com (D.H.Y.); serkin@korea.edu (S.I.P.); 3 Department of Urology, College of Medicine, Chung-Ang University, Seoul 06974, Korea; mir0302@naver.com; 4 College of Pharmacy, Chung-Ang University, Seoul 06974, Korea; phantomryda@naver.com

**Keywords:** AMP activated protein kinase (AMPK), reactive oxygen species (ROS), endoplasmic reticulum (ER) stress, bladder cancer, orthotopic bladder cancer mouse model, BCG-CWS drug-delivery system

## Abstract

**Simple Summary:**

We engineered novel nanoparticles consisting of liposome-encapsulated Bacillus Calmette–Guérin cell well skeleton (BCG-CWS) for intravesical instillation in bladder cancer. The liposome-encapsulated BCG-CWS nanoparticles had antitumoral effects in an orthotopic bladder cancer mouse model, and the BCG-CWS nanoparticles can be further developed as a non-toxic substitute for live BCG with improved dispensability, stability, and size compatibility. This is significant because we succeeded in the intravesical delivery of BCG-CWS through the intravesical route using a catheter in an orthotopic bladder cancer mouse model to specifically target tumor cells. This is the first study on the BCG-CWS-induced activation of AMPK in urothelial carcinoma cells, suggesting that AMPK-mediated reactive oxygen species (ROS) production and ER stress is a cellular signaling pathway in tumors sensitive to BCG-CWS. These results have the potential for significant ramifications in targeted therapy using a predictive marker for bladder cancer.

**Abstract:**

The *Mycobacterium* Bacillus Calmette-Guérin cell wall skeleton (BCG-CWS), the main immune active center of BCG, is a potent candidate non-infectious immunotherapeutic drug and an alternative to live BCG for use against urothelial carcinoma. However, its application in anticancer therapy is limited, as BCG-CWS tends to aggregate in both aqueous and non-aqueous solvents. To improve the internalization of BCG-CWS into bladder cancer cells without aggregation, BCG-CWS was nanoparticulated at a 180 nm size in methylene chloride and subsequently encapsulated with conventional liposomes (CWS-Nano-CL) using an emulsified lipid (LEEL) method. In vitro cell proliferation assays showed that CWS-Nano-CL was more effective at suppressing bladder cancer cell growth compared to nonenveloped BCG-CWS. In an orthotopic implantation model of luciferase-tagged MBT2 bladder cancer cells, encapsulated BCG-CWS nanoparticles could enhance the delivery of BCG-CWS into the bladder and suppress tumor growth. Treatment with CWS-Nano-CL induced the inhibition of the mammalian target of rapamycin (mTOR) pathway and the activation of AMP-activated protein kinase (AMPK) phosphorylation, leading to apoptosis, both in vitro and in vivo. Furthermore, the antitumor activity of CWS-Nano-CL was mediated predominantly by reactive oxygen species (ROS) generation and AMPK activation, which induced endoplasmic reticulum (ER) stress, followed by c-Jun N-terminal kinase (JNK) signaling-mediated apoptosis. Therefore, our data suggest that the intravesical instillation of liposome-encapsulated BCG-CWS nanoparticles can facilitate BCG-CW cellular endocytosis and provide a promising drug-delivery system as a therapeutic strategy for BCG-mediated bladder cancer treatment.

## 1. Introduction

Although urothelial carcinoma is more prevalent in the United States and other Western countries than in Asian countries, the incidence of bladder cancer in Korea has increased two-fold from 101,032 cases in 1999 to 218,017 cases in 2011 [1]. With this increase in bladder cancer incidence, there is an increased need for an effective drug-delivery system targeting non-muscle-invasive bladder cancer (NMIBC); the intravesical instillation of Bacillus Calmette–Guérin (BCG) is one such promising system. BCG is currently the standard immunotherapeutic drug for bladder cancer; it has beneficial clinical effects through the induction of the non-specific immune response [2,3]. However, the intravesical use of live BCG can induce side effects due to infections, even if these events are reported to have a lower than 5% incidence [4,5]. To overcome the complications following BCG instillation, BCG cell wall skeleton (BCG-CWS) has been used as a safe and effective alternative to live BCG. BCG-CWS has been isolated as the main immune active component of BCG and is used as a potent immunoadjuvant for cancer immunotherapy [6,7,8]. The use of BCG-CWS as an adjuvant in cancer immunotherapy has shown promising results in patients for many years [8,9]. To apply intravesical BCG-CWS instillation into the bladders of animals and humans, the development of an appropriate delivery system has been required, as its large size and insolubility in both aqueous and organic solvents limit its use [10]. Therefore, it has been a challenge to develop a formulation that disperses uniformly without forming aggregates in an aqueous environment [11]. We recently reported an intracellular delivery system for BCG-CWS involving liposome-encapsulated BCG-CWS nanoparticles that circumvented the limitations of the intravesical instillation of BCG-CWS in bladder cancer [12].

The biologically active components of BCG-CWS include mycolic acids, arabinogalactan, and peptidoglycan; they have the potential to be used as immune adjuvants in cancer immunotherapy [6,13]. Several studies have reported the antitumor activity of BCG-CWS; it has been shown to elicit an antigen-specific cellular immune response through toll-like receptors (TLR-2 and TLR-4) when co-administered with tumor antigens [14,15]. Despite the regular clinical use of BCG-CWS to regress tumor growth in cancer patients as well as in mice [6,10], the pathways involved in the associated cellular downstream signaling and host inflammatory response to BCG-CWS remain largely unknown. In a study that demonstrated the radiosensitizing effect of BCG-CWS, the authors showed that CWS induced caspase-independent apoptosis in a CT116 colon carcinoma, which enhanced the ionizing radiation (IR)-mediated autophagy and generation of ROS through the JNK pathway [16]. We previously reported that the BCG-induced innate immune response can be blocked by a mitogen-activated protein kinase (MAPK) inhibitor, which enhances the half-life of BCG by reducing the release of antimicrobial peptides by TLR-2 activation. Additionally, MAPK activation, stimulated by TLR-2 or TLR-4 agonists in mice, can be inhibited by an AMPK activator [17]. Therefore, we hypothesized that the antitumor activity of BCG-CWS may be attributed to ROS-stimulated cellular stress such as the activation of AMPK and ER stress pathways. A recent study reported that *Mycobacterium tuberculosis* in macrophages induces protease-activated receptor-4 (PAR-4) production through ER stress, which is involved in the blocking of the conversion of the autophagy marker LC3B-I to LC3B-II, thereby triggering apoptosis through the suppression of protective autophagy [18]. In this study, we demonstrate that although treatment with BCG-CWS stimulates the initiation of autophagy-related proteins, it does not lead to the formation of autophagosomes to induce apoptosis. Our findings also show that the antitumor activity of BCG-CWS induces ROS production and AMPK activation, which elicits JNK signaling-mediated apoptosis, owing to the stimulation of the ER stress pathway such as the phosphorylation of both inositol-requiring enzyme 1α (IRE1α) and PAR-4. Notably, we demonstrate that liposome-encapsulated BCG-CWS nanoparticles could enhance the delivery of BCG-CWS in an orthotopic bladder cancer mouse model.

## 2. Results

### 2.1. Physical Characteristics of CWS-Loaded Formulations

**Figure 1:**
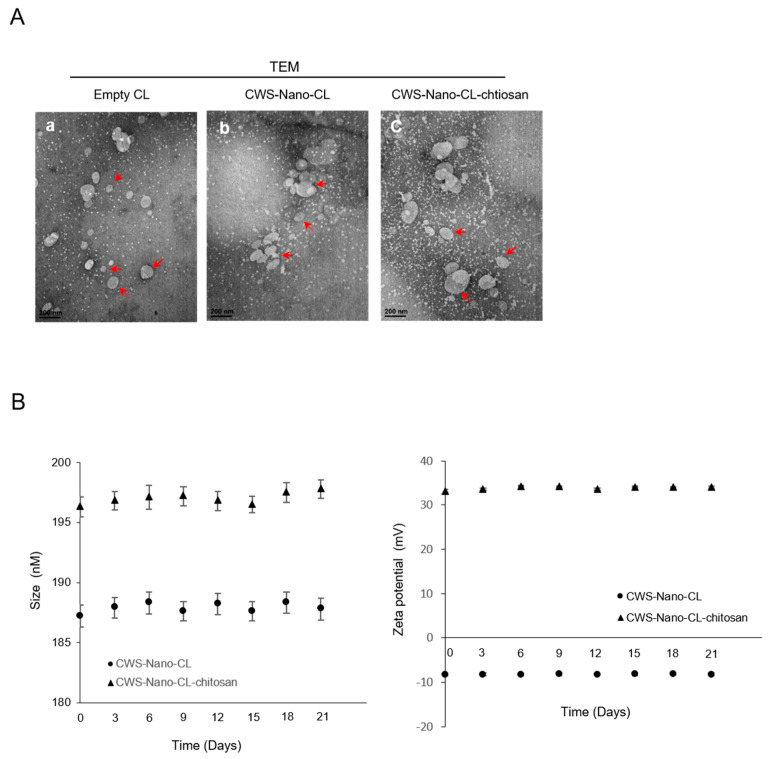
Characterization of the CWS-loaded formulations. (**A**) Transmission electron microscopy (TEM) images of empty CL, CWS-Nano-CL, and CWS-Nano-CL-chitosan. The red arrows indicate the prepared liposomes. All of the scale bars indicate 200 nm. (**B**) Colloidal stability of the CWS-Nano-CL, and CWS-Nano-CL-chitosan. Data are mean ± SD (*n* = 3).

Two types of CWS-loaded formulations were prepared: CWS-Nano-CL and chitosan-coated CWS-Nano-CL (CWS-Nano-CL-chitosan). Both CWS-loaded formulations were characterized as being free of aggregates and displayed excellent physical and colloidal stabilities during the experiments. As listed in Table 1, the size of the prepared liposomes was 187 nm for the CWS-Nano-CL. However, the size of the CWS-Nano-CL-chitosan increased to 196 nm, suggesting the formation of a coating layer on the surface of the liposomes. Both liposomes exhibited a polydispersity index (PDI) value <0.2, indicating a homogenous dispersion. In terms of the zeta potential (ZP), the CWS-Nano-CL exhibited a negative value of −8 mV, whereas the CWS-Nano-CL-chitosan was inverted to a positive value (33.17 mV), representing the presence of a positively charged chitosan layer on the liposomal surface. The chitosan coating efficiency was 86%, evaluated via the ultrafiltration method. Both types of CWS-loaded formulations had an entrapment efficiency (EE) and drug loading (DL) of ~60% and ~219.87 µg/mg, respectively. Overall, the CWS encapsulation did not alter the general physical characteristics of the liposomes. As shown in Figure 1, the TEM images revealed no differences between the liposomal samples, with a size of less than 200 nm, and the colloidal stability of the liposomes was maintained for 3 weeks.

### 2.2. CWS-Loaded Formulations Inhibit the Growth of Bladder Cancer Cells

**Figure 2:**
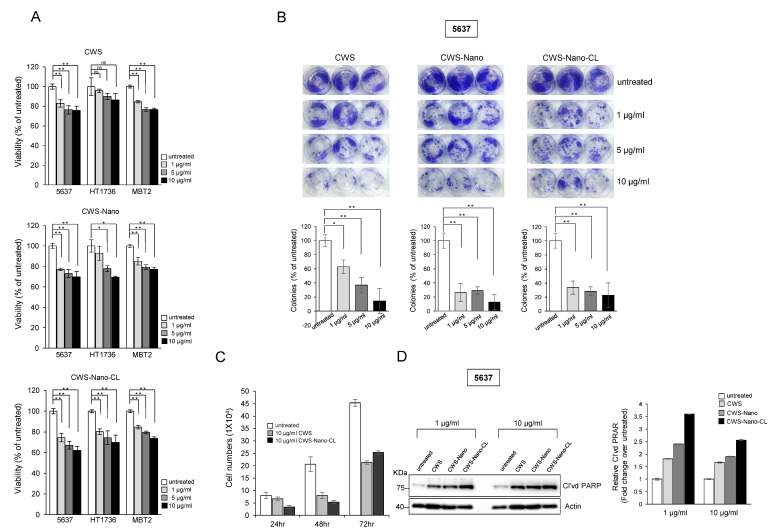
Effects of CWS-loaded formulation on growth inhibition in bladder cancer cells. (**A**) Human bladder cancer 5637 and HT1376 cells, and murine bladder cancer MBT2 cells were seeded in 96-well plates. Cells were treated with indicated concentrations of CWS-loaded formulations for 48 h, and then, cell viability was determined using MTT solution. * *p* < 0.005, ** *p* < 0.0005, ns: non-significant; untreated/CWS, untreated/CWS-Nano, or untreated/CWS-Nano-CL. Data are mean ± SEM (*n* = 6). (**B**) Colony-forming ability of cells treated with CWS-loaded formulations (1, 5, and 10 µg/mL) was measured after 2 weeks. * *p* < 0.005, ** *p* < 0.0001; untreated/CWS, untreated/CWS-Nano, or untreated/CWS-Nano-CL. Data are mean ± SEM (*n* = 3). (**C**) Cell numbers were counted by staining with trypan blue at 24, 48, and 72 h after treatment with CWS-loaded formulation. * *p* < 0.05, ** *p* < 0.0005; untreated/CWS or untreated/CWS-Nano-CL. Data are mean ± SEM (*n* = 3). (**D**) Levels of cleaved poly(ADP-ribose) polymerase (PARP) in cells treated with CWS-loaded formulations were analyzed by Western blotting. Actin was used as a loading control. The blots are representative of three independent experiments. The quantification graphs represent cleaved PARP (Cl’vd PARP)/Actin ratios determined by densitometric analyses. All expression ratios were normalized to the untreated group.

To compare the inhibitory effects of the different CWS-loaded formulations on the growth of bladder cancer cells, we first analyzed the viability of cells incubated for 48 h with various concentrations of CWS-loaded formulations (Figure 2A). The viability of the 5637 cells was reduced by 20% upon treatment with 10 µg/mL of CWS, whereas growth inhibition of 30–40% was observed when the cells were treated with the same concentration of CWS-Nano-CL. The growth of the HT1376 cells was more sensitive to treatment with CWS-Nano-CL than treatment with CWS, both used at a concentration of 10 µg/mL, whereas the MBT2 cells showed similar inhibition of growth upon treatment with all the CWS-loaded formulations. CWS-Nano-CL at a low concentration (1 µg/mL) was more effective at reducing the colony-forming ability of the 5637 cells than CWS and CWS-Nano at low concentrations (Figure 2B). All of the CWS-loaded formulations at a high concentration of 10 µg/mL completely abolished the colony-forming ability of the 5637 cells. To test the effect of the CWS-loaded formulations on cell growth, the surviving cells were counted post-treatment via trypan blue exclusion at 24, 48, and 72 h (Figure 2C). Consistent with the effect observed on colony formation, CWS-Nano-CL was the most effective at inhibiting cell growth. Similarly, the cleavage of poly(ADP-ribose) polymerase (PARP) in the 5637 cells treated with CWS-Nano-CL was evidently more enhanced than that in the cells treated with CWS or CWS-Nano (Figure 2D). These results indicate that the drug-delivery system involving nanoparticulation and encapsulation with liposomes enhanced the growth inhibition of bladder cancer cells similarly to treatment with plain BCG-CWS.

### 2.3. CWS-Loaded Formulations Inhibit the mTOR Pathway and Induce the Initiation of Autophagy through AMPK Activation

**Figure 3:**
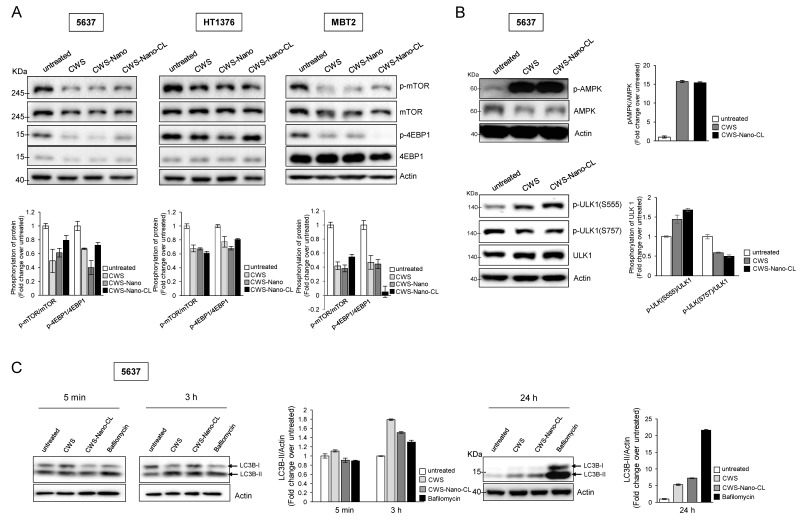
Induction of autophagy initiation by CWS-loaded formulations via mTOR signaling inhibition and AMPK activation in bladder cancer cells. (**A**) Cells were treated with 1 μg/mL of CWS-loaded formulations for 24 h, and the lysates were subjected to Western blotting. (**B**) 5637 cells were treated with 1 μg/mL of CWS or CWS-Nano-CL for 24 h, and then, the phosphorylated AMPK to total AMPKα protein expression ratio was assessed by Western blotting. As they are autophagy-initiation-related proteins, the ratio of phosphorylated ULK1 (Ser555 and Ser757) to total ULK protein expression was detected by Western blotting. (**C**) Cells were treated with 1 μg/mL of CWS, CWS-Nano-CL, or 0.1 µM bafilomycin (Bafilo) for 5 min, 3 h, or 24 h, and the cell lysates were subjected to Western blot analysis using anti-LC3B-I/II antibodies. Actin was used the loading control. The blots are representative of three independent experiments. The quantification graphs represent p-mTOR/mTOR, p-4EBP1/4EBP1, p-AMPK/AMPK, pULK1(Ser555)/ULK1, pULK1(Ser757)/ULK1, and LC3B-II/Actin ratios determined by densitometric analyses. All expression ratios were normalized to the untreated group.

Previous reports suggest that the combination of BCG-CWS and IR induces the autophagy of colon cancer cells; moreover, this effect is reduced in colon cancer cells with a knockdown of Beclin 1 or Atg7 [16]. Since the mTOR pathway serves as a major autophagy regulator [19], we tested whether the CWS-loaded formulations could inhibit the activation of mTOR signaling in bladder cancer cells. As shown in Figure 3A, all of the CWS-loaded formulations reduced the phosphorylation of mTOR and subsequently inhibited the phosphorylation of 4EBP-1, the downstream effector of mTOR signaling. In particular, AMPKα can inhibit mTOR signaling via the phosphorylation of tuberous sclerosis complex 2 (TSC2) at Ser1387 [20]. We observed that both CWS and CWS-Nano-CL induced the phosphorylation of AMPK in the 5637 cells (Figure 2B) and other bladder cancer cells (Supplementary Figure S1), leading to a change in the autophagy-related proteins. As shown in the lower panel of Figure 3B, the Unc-51-like autophagy activating kinase 1 (ULK1) complex was regulated by treatment with CWS and CWS-Nano-CL, where activated AMPK induced the phosphorylation of ULK1 at Ser555, whereas the downregulated mTOR complex 1 suppressed its phosphorylation at Ser757. Interestingly, CWS and CWS-Nano-CL facilitated the conversion of LC3B-I to LC3B-II in the 5637 cells after 3 and 24 h, respectively, in the presence of bafilomycin A1, a late-phase autophagy inhibitor (Figure 3C). This increase in the levels of LC3B-II induced by CWS and CWS-Nano-CL does not significantly induce apoptosis, as lysosomal degradation is prevented. These results indicate that the CWS-loaded formulations can downregulate mTOR signaling and induce AMPK activation, which leads to the induction of autophagy initiation, owing to the phosphorylation of the ULK1 complex and the increase in the levels of LC3B-II cleavage, facilitating the inhibition of cell viability.

### 2.4. CWS-Loaded Formulations Increase ROS Accumulation and ER Stress through AMPK Activation in Bladder Cancer Cells

**Figure 4:**
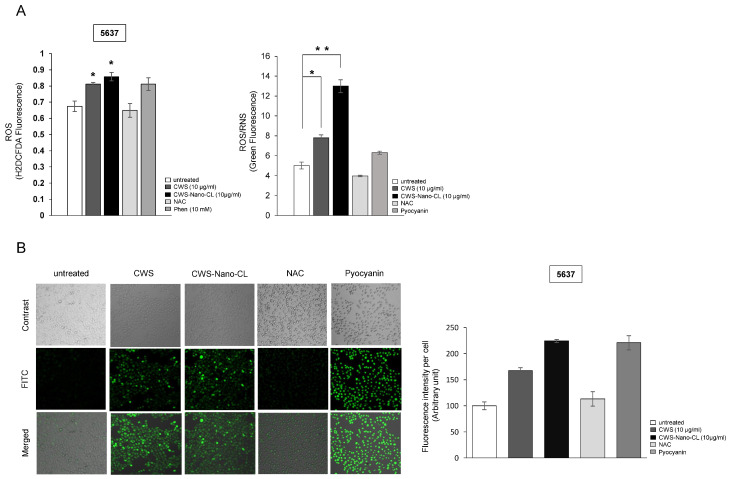
Effect of CWS-loaded formulations on reactive oxygen species (ROS) production in bladder cancer cells. (**A**) Cells were treated with 10 μg/mL of CWS or CWS-Nano-CL for 24 h and then treated with NAC (2 mM, positive control) for 30 min. After washing, cells were treated with H_2_DCFDA (10 µM) for 1 h prior to measurement. ROS/RNS production was measured using a ROS-ID^®^ ROS/RNS detection kit. Pyocyanin (500 µM) and NAC (5 mM) were added as a positive and a negative control for 30 min. * *p* < 0.005, ** *p* < 0.0005; untreated/CWS or untreated/CWS-Nano-CL. Data are mean ± SEM (*n* = 6). (**B**) Fluorescence images of ROS/RNS production in live cells were taken using a confocal microscope (Leica DMI8) and quantified with the ImageJ software program (lower panel). Data are mean ± SEM (*n* = 3).

The role of autophagy in the regulation of AMPK/mTOR signaling as a response to the CWS-loaded formulations appeared to be insufficient to inhibit cell viability and induce the apoptosis of bladder cancer cells. Previously, we showed that AMPK-mediated mTOR signaling inhibition induced aberrant ROS accumulation, causing defects in the oxidative machinery and mitochondrial membrane potential in lung and bladder cancer cells [21,22]. To explore the role of CWS-loaded formulation-induced AMPK activation in the production of ROS, we investigated whether the CWS-loaded formulations could induce ROS accumulation in bladder cancer cells. The 5637 cells labeled with carboxy-H2DCFDA, a ROS indicator dye, clearly showed the induction of ROS production after being treated with CWS-loaded formulations (Figure 4A, left panel). The levels of ROS/reactive nitrogen species (RNS) were elevated more significantly in cells treated with CWS-Nano-CL than in those treated with CWS (Figure 4A, right panel). Pyocyanin, a secondary metabolite with the ability to oxidize, was used as a positive control and a standard for ROS/RNS production. Pyocyanin-induced cells incubated with the ROS inhibitor N-acetyl L-cysteine (NAC) showed a reduction in the ROS/RNS signal compared to the untreated control cells. ROS accumulation was also observed in the HT1376 cells treated with CWS or CWS-Nano-CL, accompanied by an increase in the levels of ROS/RNS (Supplementary Figure S2). Furthermore, the fluorescence images of the 5637 cells treated with CWS or CWS-Nano-CL indicated a similar increase in ROS generation (Figure 4B).

**Figure 5:**
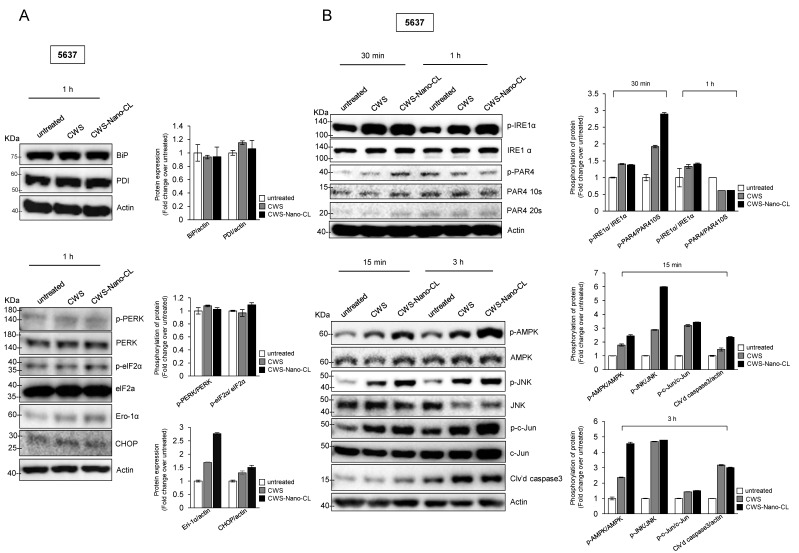
Effect of CWS-loaded formulations on ER stress-induced apoptosis via AMPK activation in bladder cancer cells. (**A**) Cells were treated with 1 μg/mL of CWS or CWS-Nano-CL for 1 h, and then, ER stress-adaptive proteins (Bip and PDI) were detected by Western blotting (left panel). ER stress-induced mediating proteins were determined by the expression of phosphorylated PERK/PERK, phosphorylated elf2α/elf2α, Ero-1α, and CHOP (right panel). (**B**) Cells were treated with 1 μg/mL of CWS or CWS-Nano-CL for 30 min and 1 h, and then, phosphorylated IRE1α/IRE1α and phosphorylated PAR4/PAR4, ER stress-inducing apoptotic proteins, were detected by Western blotting (left panel). After 15 min and 3 h of treatment, the lysates were analyzed by Western blotting using specific antibodies for the indicated proteins (right panel). CWS- and CWS-Nano-CL-induced AMPK activation mediated JNK–c-Jun-caspase 3 signaling downstream of IRE1α. The blots are representative of three independent experiments. The quantification graphs represent BIP/Actin, PDI/Actin, p-PERK/PERK, p-eIF2α/eIF2α, Ero-1α/Actin, CHOP/Actin, p-IRE1α/IRE1α, p-PAR4/PAR410S, p-AMPK/AMPK, p-JNK/JNK, p-c-Jun/c-Jun, and Cl’vd PARP/Actin ratios determined by densitometric analyses. All expression ratios were normalized to the untreated group.

It has been reported that ER stress can be regulated by AMPK, which stimulates the assembly of the pre-autophagosomal structure and progression of autophagosome formation [23,24]. Since our results showed that AMPK activation by the CWS-loaded formulations induced autophagy, we next probed the effects of the CWS-loaded formulations on ER stress-related signaling in the 5637 cells. The expression levels of BiP (an immunoglobulin heavy chain-binding protein, also referred to as glucose-regulated protein-78, GRP78) and PDI (protein disulfide isomerase, an ER stress-adaptive protein did not change upon CWS and CWS-Nano-CL treatment in the 5637 cells (Figure 5A, left panel). However, CWS and CWS-Nano-CL activated the ER stress-induced mediating proteins phosphorylated eukaryotic initiation factor 2α (elF2α) and endoplasmic reticulum oxidoreductin-1α (Ero-1α) (Figure 5A, right panel). We then hypothesized that the increased ROS production with CWS and CWS-Nano-CL resulted in ER stress, leading to the induction of apoptosis. As shown in Figure 5B, CWS and CWS-Nano-CL increased the phosphorylation of both IRE1α and PAR-4, leading to apoptosis due to prolonged ER stress (Figure 5B; left panel). Moreover, it has been suggested that ER stress-activated JNK signaling induces apoptosis by the formation of the TRAF2–ASK1–IRE1 complex by IRE1α cytosolic kinase [25]. Consistent with this, CWS and CWS-Nano-CL increased the phosphorylation of JNK and subsequently activated the downstream factor c-Jun, followed by the cleavage of caspase 3 (Figure 5B, right panel). We confirmed that this cascade induced AMPK activation in the presence of CWS and CWS-Nano-CL (Figure 5B, right panel). These results suggest that CWS and CWS-Nano-CL induce the IRE1α-JNK-caspase 3 pathway as an apoptotic response to ER stress, which is mediated by AMPK activation and ROS generation in bladder cancer cells treated with CWS or CWS-Nano-CL.

### 2.5. CWS-Loaded Formulations Induce Tumor Regression in an Orthotopic Bladder Cancer Mouse Model

**Figure 6:**
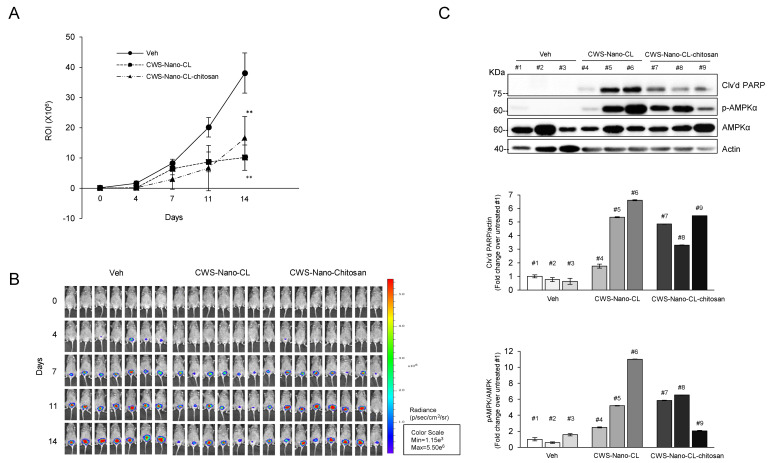
Effects of CWS-loaded formulations on tumor regression in an orthotopic bladder cancer mouse model. (**A**,**B**) Luciferase gene-expressing MBT2 (MBT2-Luc) cells were implanted inside the bladder of C3H/He mice. MBT2-Luc tumors in the bladders of C3H mice were detected by IVIS (Perkin Elmer, Waltham, MA, USA). Upon the luminescent intensity being captured via BLS, Veh, CWS-Nano-CL, or CWS-Nano-CL-chitosan were instilled through a catheter into the bladder lumen and remained in situ for 2 h. In all of the statistical analyses, two-sided p-values less than 0.05 were considered statistically significant. ROI time plots for quantitative comparison (Veh/CWS-Nano-CL, Veh/CWS-Nano-CL-chitosan; ** *p* < 0.005). Data represent the means ± SD (*n* = 7). (**C**) CWS-Nano-CL and CWS-Nano-CL-chitosan induced cleaved PARP by activation of AMPK in orthotopic bladder cancer mice. The blots are representative of three independent experiments. All of the mice were sacrificed, and whole bladders of mice were collected from them. Western blot analysis was performed on the bladder tissues of C3H/He mice, and each bladder tumor number is labeled on top o the bands. The blots are representative of three independent experiments. The quantification graphs represent Cl’vd PARP/Actin and p-AMPK/AMPK ratios determined by densitometric analyses. All expression ratios were normalized to the untreated group. Abbreviations: Veh, Vehicle; CWS-Nano-CL, CWS nanoparticles encapsulated with conventional liposomes; CWS-Nano-CL-chitosan, chitosan-coated CWS-Nano-CL; BLS, bioluminescence signal; ROI, region of interest.

Collectively, our in vitro data demonstrate that CWS or the CWS-loaded formulations inhibited the growth of bladder cancer cells through the activation of AMPK, suggesting that the anticancer effect of CWS and CWS-Nano-CL may be due to the induction of both ROS and the ER stress pathway. The insolubility of CWS in hydrophilic and hydrophobic solvents limits its use. The intravesical administration of CWS via a catheter shows limited antitumor efficiency in vivo. Therefore, to test the antitumor efficacy of CWS-loaded formulations in an animal model, CWS-Nano-CL and CWS-Nano-CL-chitosan (chitosan-coated CWS-Nano-CL) were prepared because two types of CWS-loaded liposomes were characterized as being free of aggregates (Table 1). CWS-Nano-CL-chitosan had a mucoadhesive property, improving drug concentrations at targeted sites. To establish an orthotopic bladder cancer mouse model, luciferase gene-expressing MBT2 (MBT2-Luc) cells were used to inoculate the bladder of C3H/He mice. The vehicle, CWS-Nano-CL, or CWS-Nano-CL-chitosan was then instilled into the bladder lumens of the mice using an in situ catheter for 2 h to maximize the internalization of the CWS-loaded formulations into the MBT2-Luc tumors in the bladders of the C3H/He mice. In vivo bioluminescence imaging showed that the intravesical CWS-loaded formulation-instilled mice had tumors of significantly reduced size. After Days 11 and 14, statistically significant differences in terms of tumor-suppressive effects were observed between the CWS-loaded formulation-treated and vehicle groups (Figure 6A,B). To confirm that the CWS-loaded formulations also induced tumor regression via AMPK activation in the mouse model, the Western blotting of samples from the mice tumors was performed at the termination of the experiment. Consistent with the in vitro data, the CWS-Nano-CL-treated tumors exhibited an induction of PARP cleavage and an increase in the levels of AMPK phosphorylation compared to the tumors in the vehicle group (Figure 6C). Additionally, the CWS-Nano-CL-chitosan-treated tumors also showed an induction of PARP cleavage and phosphorylation of AMPK, despite the fact that the tumor regression induced by the chitosan-coated liposomes was not greater than that induced by CWS-Nano-CL. Thus, our results suggest that both CWS-Nano-CL and CWS-Nano-CL-chitosan significantly reduced tumor growth in an orthotopic bladder cancer mouse model by inducing apoptosis through AMPK activation. Therefore, this approach can be used to overcome the limitations of the intravesical instillation of CWS.

## 3. Discussion

The clinical applications of homogenous BCG-CWS formulations are limited despite the fact that they display promising antitumor efficacy that can be used in adjuvant chemotherapy. The BCG-CWS-mediated induction of the innate immune system through various pattern recognition receptors has been extensively studied [26,27,28], but its effects on other cellular signaling pathways and the underlying molecular mechanisms in bladder cancer remain elusive. Our data demonstrated that all of the CWS-loaded formulations, including CWS, inhibited the growth of bladder cancer cells through mTOR inhibition and AMPK activation, accompanied by ROS accumulation and prolonged ER stress, to induce apoptosis. Previous reports showed that BCG-CWS and IR in combination enhanced the sensitivity of colon cancer cells to radiotherapy by the induction of autophagy and ROS generation [16]. Consistent with this report, our data showed that CWS and the CWS-loaded formulations induced the phosphorylation of the ULK1 complex, initiation of autophagy, and accumulation of ROS (Figure 3B). Recently, we reported that an mTOR inhibitor induced the initiation of autophagy, but it was not adequate to enhance cell death, owing to the protective effect of NBR1, an antioxidant-related gene, in bladder cancer cells [22]. In addition, other reports suggest that, under normal conditions, basal autophagy maintains homeostasis for cell viability by interacting with apoptosis repressors such as Bcl-2 or Bcl-xL, whereas, under stress conditions, amplified autophagy has a prosurvival role [29]. According to our data, CWS and CWS-Nano-CL stimulated AMPK- and mTOR-dependent autophagy, which was inefficient in inducing apoptosis, owing to the increased conversion of LC3B-II, which can prevent autophagosome–lysosome fusion and LC3B-II degradation (Figure 3C). Autophagy is upregulated in response to extracellular and intracellular stress and signals such as starvation, growth factor deprivation, ER stress, and pathogen infections [30]. Therefore, we hypothesized that the CWS- and CWS-loaded formulation-induced cell death may be associated with prolonged ER stress due to the generation of ROS. Our data demonstrated that CWS-Nano-CL greatly induced ROS accumulation (Figure 4A,B), leading to the induction of ER stress-mediated apoptotic proteins (Figure 5A). More importantly, we found that AMPK activation by CWS or CWS-Nano-CL induced the phosphorylation of both IRE1α and PAR-4, which subsequently led to JNK signaling-mediated apoptosis (Figure 5B).

Although the classical MAPK signaling pathway promotes survival, protecting against chemotherapeutic agents, recent studies provide new insights into the function of the p38 and JNK/MAPK pathways in the maintenance of the balance between autophagy and apoptosis in response to ER stress [31]. p38 regulates this balance through the PERK/eukaryotic initiation factor 2α (eIF2α)–AEF4 and IRE1–JNK1 pathways [32,33]. Additionally, the increased PAR-4 expression observed in the 5637 cells treated with CWS or CWS-Nano-CL is associated with apoptosis. PAR-4, which is an apoptotic protein identified in prostate cancer cells undergoing apoptosis, is sufficient to induce apoptosis in most cancer cells [34]. Burikhanov et al. demonstrated that extracellular PAR-4 induced apoptosis by binding to GRP78, the stress response protein expressed on the surface of most cancer cells [33]. In our data, BiP (or GRP78) was highly expressed in the 5637 bladder cancer cells (Figure 5a). Moreover, Han et al. showed that, in prostate cancer cells treated with BCG, there was an increase in PAR-4 activation as a defense against mycobacteria, which in turn led to ER stress-induced apoptosis through intracellular ROS generation and the activation of caspases [18]. Collectively, previous data from other groups consistently indicate that various cellular mechanisms such as ER stress, AMPK activation, and ROS generation can induce apoptosis in cancer cells. Our observations also indicate that similar mechanisms may be responsible for conferring sensitivity to CWS or CWS-Nano-CL.

Importantly, in this study, we developed an improved system for the intravesical delivery of BCG-CWS in an orthotopic tumor mouse model of bladder cancer. Previously, we performed flow cytometry and a confocal laser scanning microscopy assay to evaluate the cellular uptake of CWS-Nano-CL in 5637 and MBT2 cells [12]. We showed that the fluorescence peak shift (MFI) value was 34.95 according to flow cytometry, and a strong fluorescence was visualized by confocal laser scanning microscopy (CLSM) for 2 h. As for the results, the cellular uptake of CWS-Nano-CL in MBT2 cells was clearly demonstrated, indicating internalized fluorescence within the cytoplasm for 2 h. To observe the in vivo antitumor efficacies, MBT2-Luc cells were instilled into the bladders of C3H/HeN mice. CWS-Nano-CL and CWS-Nano-CL-chitosan showed statistically significant tumor regression, which resulted in AMPK activation and apoptosis (Figure 6A,C). Although BCG-CWS is a potent candidate non-infectious immunotherapeutic drug that could serve as an alternative to live BCG [6,8,9], its aggregation in both aqueous and non-aqueous solvents limits its efficient use in anticancer therapy. To overcome this issue of insolubility and improve the internalization of BCG-CWS into bladder cancer cells, we encapsulated it within a liposomal nanoparticle using an emulsification-solvent evaporation method [12,35]. We showed that, in vitro, though both CWS and CWS-Nano-CL showed antitumor effects on bladder cancer cells, the latter displayed greater efficiency than the former. Subsequently, we confirmed that both CWS and CWS-Nano-CL induced the inhibition of mTOR and activation of AMPK. Additionally, bladder cancer cells treated with CWS or CWS-Nano-CL exhibited multiple stress responses such as the initiation of autophagy, the generation of ROS, and ER stress. Previously, Nakamura et al. demonstrated that a 166 nm nanoparticle encapsulating BCG-CWS instilled into the bladders of rats bearing the N-butyl-N-(4-hydroxybutyl)-nitrosamine (BBN)-induced urinary bladder cancer induced significant tumor regression [10]. This nano-structure formulation of encapsulated BCG-CWS encouraged us to administer CWS-Nano-CL through the intravesical route in an orthotopic bladder cancer mouse model. As shown in Figure 6A, our formulations of CWS-Nano-CL and CWS-Nano-CL-chitosan were successfully instilled into the bladders of the orthotopic bladder cancer model mice without the formation of hydrophobic aggregates of CWS. As this is distinguishable from the bladder cancer cell-xenograft rat model of Nakamura’s study, we demonstrated for the first time that CWS-Nano-CL and CWS-Nano-CL-chitosan could be injected into the bladder via a catheter in an orthotopic bladder cancer mouse model. Moreover, to enhance the mucoadhesion of CWS-Nano-CL to the bladder surface of the model mice, chitosan-coated liposomes were developed. Despite the added function of mucoadhesion, both CWS-Nano-CL and CWS-Nano-CL-chitosan exhibited similar tumor regression induction. Thus, CWS-Nano-CL is a functionalized liposome encapsulating BCG-CWS that can be intravesically instilled into orthotopic bladder cancer mice to specifically target tumor cells.

Because the bladder is one of the few organs in which agents can be delivered directly to a targeted tumor site, applying the intravesical instillation of BCG-CWS would clearly be beneficial. Thus, to better prevent aggregation issues with BCG-CWS, the insertion of a catheter into the urethra for the delivery of BCG-CWS is used. Recently, the development of a new packaging method has allowed BCG-CWS to be applicable as a bladder cancer drug, which is optimal for the chemotherapy of bladder cancer. Additionally, the antitumor effect of nanoparticulates of BCG-CWS was associated with an increase in the internalization of BCG-CWS into bladder cancer cells, resulting in the initiation of antitumor immunological activity [36]. As another approach applied to the nanoparticulation of BCG-CWS, the use of octaarginine-modified liposomes incorporating BCG-CWS (R8-liposome-BCG-CW) exhibited an increase in cellular internalization, which induced growth inhibition in an in vivo bladder cancer model, showing the immunotherapeutic potential of BCG-CWS for NMIBC [37,38]. Moreover, co-treatment with R8-liposome BCG-CWS and BCG mediated surface specific ligands (NKG2D) in bladder cancer cells; this effect resulted in enhanced sensitivity to cytolysis by lymphokine-activated killing cells [39]. These studies indicate that the optimization and development of the encapsulation of BCG-CWS with functionalized liposomes for bladder cancer will provide significant advances for improving BCG-CWS immunotherapy against bladder cancers. According to the present study, CWS-Nano-CL and CWS-Nano-CL-chitosan can offer potential benefits for intravesical instillation providing agents directly to targeted bladder tumor cells, ultimately mediating antitumor effects with AMPK activation.

## 4. Materials and Methods

### 4.1. Cell Culture and Antibodies

The human bladder cancer 5637 cells and HT1376 cells were purchased from the American Type Culture Collection (Manassas, VA, USA). The mouse bladder cancer MBT2 cells were purchased from the Korean Cell Line Bank (Seoul, Korea). MBT2-Luc cells expressing luciferase, obtained from Dr. S.J. Lee (National Cancer Center, Gyeonggi-do, Korea) [17], were used to monitor tumor growth and responses using the non-invasive in vivo imaging of the bladder. All cells were cultured in RPMI 1640 medium supplemented with 10% FBS (Gibco Laboratories, MD, USA) and 1% penicillin–streptomycin. All cells were subcultured at 70–80% confluency and used for treatment at the 5th–20th passages. Cells were incubated at 37 °C in a humidified atmosphere containing 5% CO_2_. Rabbit polyclonal antibodies against cleaved PARP, ULK1, phospho-ULK1 (Ser757), phospho-ULK1 (Ser555), phospho-mTOR (Ser2448)/mTOR, phospho-4E-BP1 (Ser65)/4E-BP1, phospho-AMPKα (Thr172)/AMPK, BiP, phospho-IRE1α/IRE1, phospho-PAR-4/PAR-4, phospho-JNK/JNK, phospho-c-Jun (Ser63)/c-Jun, and caspase 3 were purchased from Cell Signaling Technology (Danvers, MA, USA). A rabbit polyclonal antibody against LC3B-I/II was purchased from Novus Biologicals (Littleton, CO, USA). A mouse monoclonal antibody against actin was purchased from Santa Cruz Biotechnology (Dallas, TX, USA).

### 4.2. Preparation of the CWS-Loaded Liposomal Formulations

#### 4.2.1. Preparation of CWS-Nano-CL

BCG-CWS (Pasteur 1173P2) was provided by Dr. T.H. Paik (Chungnam National University, Daejeon, Korea) and was prepared as previously described [6], with slight modifications [16]. To prepare the nanoparticles containing liposome-encapsulated BCG-CWS, CWS was encapsulated into the liposomes using the “liposome evaporated via emulsified lipid” method, with slight modifications [10,12]. For BCG-CWS encapsulation, plain liposomal vesicles composed of soy phosphatidylcholine and cholesterol (in a 9:1 molar ratio) were prepared using the thin film hydration method. Briefly, the components were dissolved in a mixture of methanol and chloroform (2:1 *v*/*v*) and subjected to rotary evaporation at 40 °C under reduced pressure. The thin film formed was then exposed to a nitrogen gas stream for 2 h and finally hydrated with 10 mM phosphate-buffered saline (PBS). Separately, 3 mg of BCG-CWS was dissolved in a methylene chloride solution and vortexed gently to obtain a homogenous dispersion. Next, 2.1 mL of liposomal solution was emulsified with 0.9 mL of CWS-Nano dispersed in the methylene chloride solution using a sonicator (Sonopuls, HD 2070; Bandelin Electronics, Berlin, Germany). After removing the solvent by rotary evaporation, the emulsion was extruded 10 times through a 200 nm membrane filter using a mini-extruder (Avanti^®^ Polar Lipids, Alabaster, AL, USA).

#### 4.2.2. Preparation of CWS-Nano-CL-Chitosan

For the preparation of the chitosan-coated liposomes (CWS-Nano-CL-chitosan), chitosan was dissolved in acetic acid (1%, *v*/*v*) and further diluted with sodium acetate buffer (pH 6.0) to obtain a chitosan concentration of 0.4%. The extruded suspension (CWS-Nano-CL) was then added dropwise to the cholesterol solution in equal volumes, with continuous stirring at 10 °C for 1 h, to form the CWS-Nano-CL-chitosan. Empty liposomes without BCG-CWS were prepared separately. All the liposomal samples were stored at 4 °C and used within 3 weeks.

#### 4.2.3. Particle Size and Zeta Potential (ZP) Analysis

The liposomal nanoparticles were diluted with distilled water and examined for their size distribution and polydispersity index (PDI) using a dynamic light scattering particle size analyzer.

#### 4.2.4. Entrapment Efficiency (EE) of the BCG-CWS Liposomal Formulations

The EE of the liposomal formulations was determined using a previously reported method [10]. Briefly, the liposomes were disrupted with ethanol and centrifuged at 3000× *g* for 5 min at 15 °C to obtain precipitated BCG-CWS. The pellet was dissolved in hexane and mixed with a 0.55% carbol-fuchsin solution. The hexane fraction was then collected, and the absorbance at 530 nm was measured using a microplate reader (FlexStation 3; Molecular Devices, Sunnyvale, CA, USA). The EE and drug loading (DL) were calculated according to the following equations:

EE (%) = (WT − WF)/WT × 100(1)

DL (μg/mg) = (WT − WF)/WL(2)

where WT, WF, and WL represent the total amount of the drug (BCG-CWS) added, the amount of free drug, and the total amount of lipid initially added, respectively.

#### 4.2.5. Chitosan Coating Efficiency

The chitosan coating efficiency of the liposome was evaluated via the ultrafiltration method as previously reported [40]. Briefly, aliquots of chitosan–liposome (0.5 mL) were passed through an ultrafiltration tube (molecular weight cut off, 10 kDa) by centrifuging at 12,000 rpm for 5 min. The amount of chitosan in the filtrate was then determined via a colorimetric method [35] using the anionic dye Cibacron Brilliant Red 3B-A, which reacts with the amino groups in cholesterol molecules. Briefly, a 0.0075% (*w*/*v*) dye solution was prepared in citrate buffered saline (pH 3.2). For analysis, 3 mL of the prepared dye solution was mixed with 0.3 mL of the filtrate obtained from ultrafiltration and then incubated in a water bath for 5 min at 30 °C. The absorbance was measured at 575 nm using a UV–visible spectrophotometer (UV-9100, Shanghai, China) to determine the coating efficiency.

#### 4.2.6. Colloidal Stability of Liposomal Formulations

Liposomal nanoparticles were stored at 4 °C for up to 3 weeks to evaluate their colloidal stability. Aliquots were withdrawn periodically and analyzed for their particle size and ZP using dynamic light scattering as described above.

#### 4.2.7. Transmission Electron Microscopy (TEM)

For observing the morphology, liposomal samples were imaged using a transmission electron microscope (JEM1010; JEOL, Tokyo, Japan) at an acceleration voltage of 80 kV. Briefly, liposomal samples were diluted with distilled water (100-fold) and placed on a carbon film grid. The samples were stained with 2% phosphotungstic acid, washed with distilled water, and dried at 25 °C prior to observation.

### 4.3. Cell Viability and Clonogenic Assays

Various concentrations (1, 5, and 10 µg/mL) of the CWS-loaded formulations were added to bladder cancer cells to analyze viability and colony formation. Cells were plated on 96-well plates (5 × 10^3^ cells/well) in complete medium and treated with the indicated concentrations of CWS-loaded formulations. After 48 h of treatment, cell viability was analyzed using the 3-(4,5-dimethylthiazol-2-yl)-2,5-diphenyltetrazolium bromide (MTT) assay according to the manufacturer’s instructions (Sigma-Aldrich, St. Louis, MO, USA). Empty liposomal formulations were analyzed for cytotoxicity using the same method. For the colony formation assay, the cells were plated on a 12-well plate (100 cells/well) containing complete medium, and the cells were allowed to attach to the plate. On the following day, the cells were treated with various concentrations (1, 5, and 10 µg/mL) of the CWS-loaded formulations and incubated for 2 weeks to allow colony formation. Before imaging, the colonies were fixed with 6% glutaraldehyde and stained with 0.1% crystal violet (Sigma-Aldrich). Colonies that were ≥50 μm in size were counted as individual clones using the ImageJ software (National Institutes of Health, Bethesda, MD, USA). For the growth curve, cells were seeded in 6-well plates at 2 × 10^4^ cells/well and treated with 10 µg/mL CWS-loaded formulations. At 24, 48, and 72 h after treatment, the trypan blue-negative surviving cells were counted under a microscope.

### 4.4. Western Blot Analysis

Cells were seeded on a 6-well plate (5 × 10^5^ cells/well) containing complete medium and treated with 10 µg/mL of the CWS-loaded formulations. After treatment, the total cell lysates were subjected to SDS-PAGE and then transferred onto polyvinylidene difluoride membranes (Millipore, Burlington, MA, USA). The transferred membranes were blocked with 5% non-fat milk in 0.1% Tween 80 and incubated overnight with the primary antibody (1:1000) at 4 °C. The membranes were washed and incubated with the secondary antibody (1:5000) for 1 h at room temperature. After washing, the membranes were analyzed using the ChemiDoc gel imaging system (Bio-Rad, Hercules, CA, USA). All experiments were performed in triplicate.

### 4.5. ROS Accumulation and ROS/RNS Production

To measure reactive oxygen species by imaging microplate assays, the 2′,7′-dichlorodihydrofluorescein diacetate (H_2_DCFDA) non-fluorescent probe was used, which oxidizes to fluorescent 2′,7′-dichlorofluorescein (DCF) under ROS exposure. Cells were seeded on a 96-well plate (5 × 10^3^ cells/well) and treated with the CWS-loaded formulations (10 µg/mL) for 24 h. After treatment, the cells were washed twice with Hank’s balanced saline solution and stained with 10 μM H_2_DCFDA dye for 1 h at 37 °C in the dark. NAC (Sigma-Aldrich) and hydrogen peroxide were used as a negative control (ROS scavenger) and positive control (intracellular ROS generator), respectively. Fluorescence was measured at excitation/emission wavelengths of 485/530 nm using the FL600 microplate reader (BioTek Instruments, Inc., Winooski, VT, USA). A ROS-ID^®^ ROS/RNS detection kit (Enzo Life Science Inc., Farmingdale, NY, USA) was used to directly monitor the ROS/RNS production in live cells according to the manufacturer’s instructions. Before ROS staining, positive and negative control samples were incubated with an ROS inducer (pyocyanin, 500 µM) and inhibitor (NAC, 5 mM) for 30 min. The plates were then washed twice and incubated with the same volume of ROS detection solution in the dark for 1 h. After all of these processes, images were captured using a Leica DMI8 confocal microscope (Leica Microsystems Inc., Buffalo Grove, IL, USA) and quantified using the ImageJ software. All experiments were performed in triplicate.

### 4.6. Animal Studies

MBT2 cells are murine bladder cancer cells with epithelial characteristics. To monitor tumor growth and the response to treatment using non-invasive in vivo imaging of the bladder, MBT-2 cells expressing luciferase (MBT-2 Luc cells) were obtained from Dr. Lee (National Cancer Center, Gyeonggi-do, Korea) [41]. To establish orthotopic bladder cancer models, 8-week-old female C3H/He mice were purchased from Orient Bio (Seongnam, Korea). A suspension of MBT-2 Luc cells (2 × 10^6^ cells/50 μL of PBS) was instilled into the bladder, and the purse-string suture was tied down for 2 h. The presence of tumors in the bladder was confirmed by luminescence analysis. After 1 week, 150 mg/kg of D-luciferin was administered intraperitoneally, and bioluminescence was detected using the IVIS Lumina XRMS In Vivo Imaging System (Perkin Elmer, Waltham, MA, USA). A dose of 1 mg/kg of CWS-Nano-CL or CWS-Nano-CL-chitosan was prepared in 50 μL of PBS and instilled into the bladder lumen via urinary catheterization. Each treatment was retained in the bladder for 2 h by tying off the orifice of the urethra. The quantitative signal intensities were calculated and are presented as regions of interest. The tumor regression after treatment with CWS-Nano-CL and CWS-Nano-CL-chitosan in the orthotopic mouse model was measured using bioluminescence imaging (BLI). The mice were randomized into the vehicle, CWS-Nano-CL, or CWS-Nano-CL-chitosan groups. Each mouse (5 mice/group) was intravesically administered PBS, CWS-Nano-CL, or CWS-Nano-CL-chitosan through a catheter, twice a week. The intravesical delivery was carried out with a dwell time of 1–2 h. Serial BLI was used to monitor bladder cancer progression every 3–4 days using an IVIS Lumina XRMS.

### 4.7. Statistical Analyses

The data acquired with the MTT, colony formation, cell number, and ROS production assays were analyzed using Student’s t-test. The Mann–Whitney U test was used to compare differences in tumor weight. In all of the statistical analyses, two-sided p-values less than 0.05 were considered statistically significant.

## 5. Conclusions

Nanoparticles comprising liposome-encapsulated BCG-CWS induce multiple stress responses, including ER stress and ROS production, leading to the inhibition of mTOR and activation of AMPK. Based on the antitumor effect of CWS-Nano-CL in the orthotopic bladder cancer mouse model and its potential as a non-toxic substitute for live BCG, these nanoparticles can be developed into a functionalized liposome-delivery system with improved dispensability, stability, and size compatibility. Thus, our BCG-CWS-delivery system is a promising and effective therapeutic strategy that can be applied clinically for the internalization of CWS for intravesical instillation in bladder cancer.

## Figures and Tables

**Table 1:** Composition and physical properties of the prepared liposomes.

Formulation Liposome Composition (mol ratio)	Empty-CL	CWS-Nano-CL	CWS-Nano-CL-chitosan
PC	90	90	90
CH	10	10	10
Physical properties			
Size (nm)	185.1 ± 0.19	187.2 ± 0.19	196.33 ± 0.34
PDI	0.11 ± 0.05	0.13 ± 0.04	0.16 ± 0.03
ZP (mV)	−8.26 ± 0.17	−8.31 ± 0.37	33.17 ± 0.24
EE (%)		60.15 ± 0.28	58.25 ± 0.31
DL		219.87 ± 4.69	217.91 ± 3.29

Data represent the mean ± SD (*n* = 3). PC, phosphatidylcholine; CH, cholesterol; PDI, polydispersity index; ZP, zeta potential; EE, entrapment efficiency; DL, drug loading; CWS, chitosan.

## Supplementary Materials

The following are available online at https://www.mdpi.com/2072-6694/12/12/3679/s1. Figure S1: Effects of CWS-loaded formulations on AMPK phosphorylation in HT1376 (A) and MBT2 (B) cells, Figure S2: Effects of CWS-loaded formulations on ROS production in HT1376 cells, Figure S3: The quantitation results of Figure 2D, Figure S4: The quantitation results of Figure 3A,B. The quantitation results of Figure 3C, Figure S5: The quantitation results of Figure 5A, The quantitation results of Figure 5B, Figure S6: The quantitation results of Figure 6C, Figure S7: Whole blot showing all the bands with molecular weightmarkers on the Western blotting.
