# Supplementary material for: Liposome-Encapsulated Bacillus Calmette–Guérin Cell Wall Skeleton Enhances Antitumor Efficiency for Bladder Cancer In Vitro and In Vivo via Induction of AMP-Activated Protein Kinase
Source: Cancers (Basel). 2020 Dec 8;12(12):3679. doi: 10.3390/cancers12123679 (PMC7762541; doi:10.3390/cancers12123679)

Supplementary Materials: Liposome-Encapsulated Bacillus Calmette–Guérin Cell Wall Skeleton Enhances Antitumor Efficiency for Bladder Cancer In Vitro and In Vivo via Induction of Amp-Activated Protein Kinase

Young Mi Whang, Da Hyeon Yoon, Gwang Yong Hwang, Hoyub Yoon, Serk In Park, Young Wook Choi, and In Ho Chang

Supplementary Fig. 1

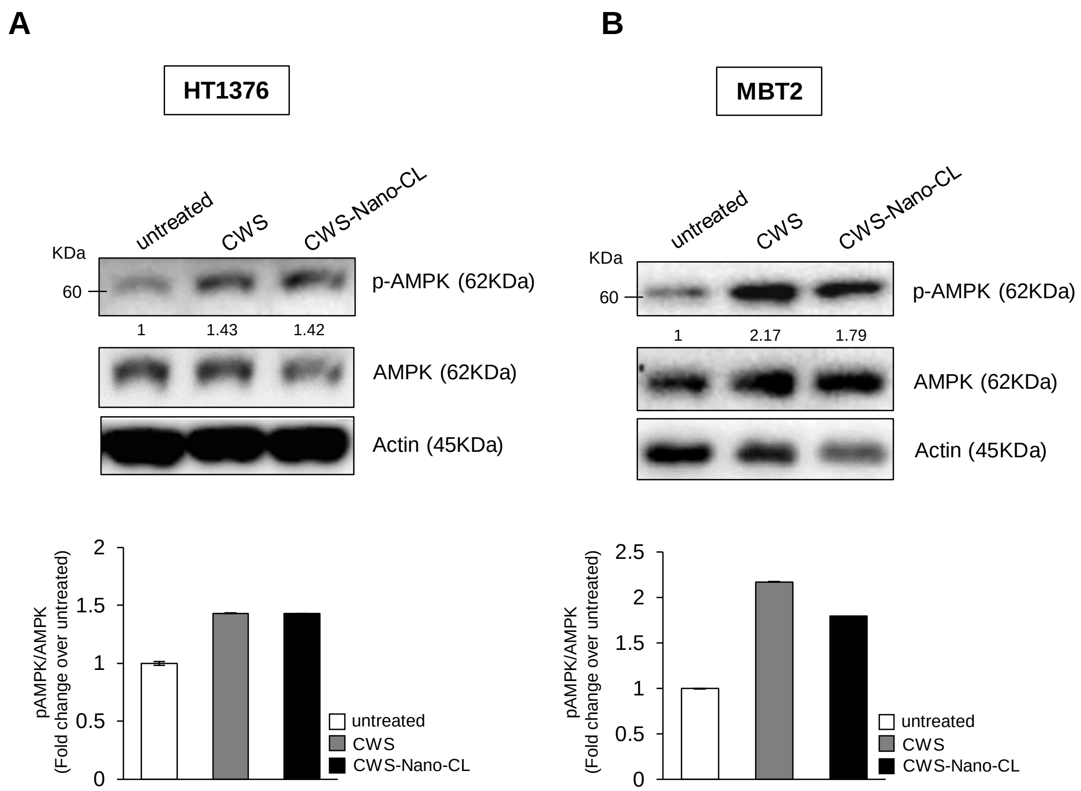

Figure S1. Effects of CWS-loaded formulations on AMPK phosphorylation in HT1376 (A) and MBT2 (B) cells**.** Cells were treated with 1 μg/ml of CWS-loaded formulations for 24 hours, and phosphorylated AMPKα to total AMPKα protein expression was assessed by western blotting. Actin was used the loading control. The blots are representative of three independent experiments. The quantification graphs are represented, p-AMPK/AMPK ratios determined by densitometric analyses. All expression ratios were normalized to the untreated group.

Supplementary Fig. 2

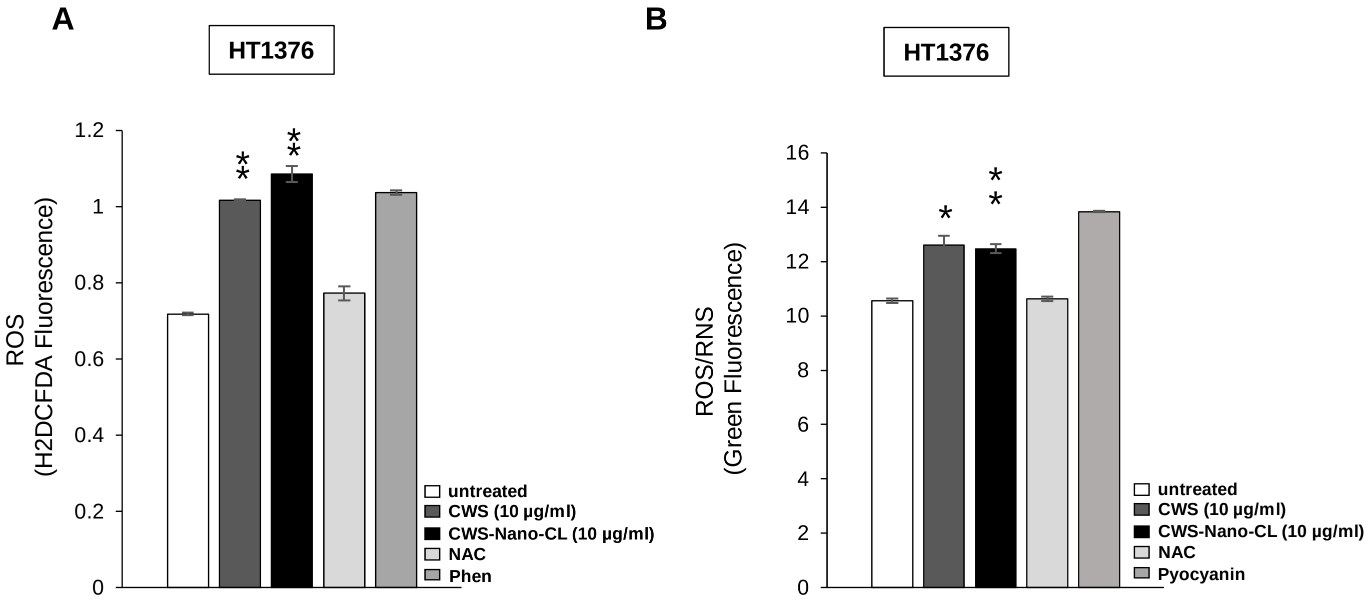

Figure S2. Effects of CWS-loaded formulations on ROS production in HT1376 cells. Cells were treated with 10 µg/ml of CWS or CWS-Nano-CL for 24 hours, then treated with NAC (2 mM, positive control) for 30 minutes. After washing, cells were treated with H2DCFDA (10 µM) for 1 hour prior to measurement. ROS/RNS production was measured using ROS-ID® ROS/RNS detection kit. Pyocyanin (500 µM) and NAC (5 mM) were added as a positive and a negative control for 30 min. **; p<0.005, **; p<0.0005*, untreated/CWS, or untreated/CWS-Nano-CL. Data are mean ± SEM (n=6).

Supplementary Fig. 3

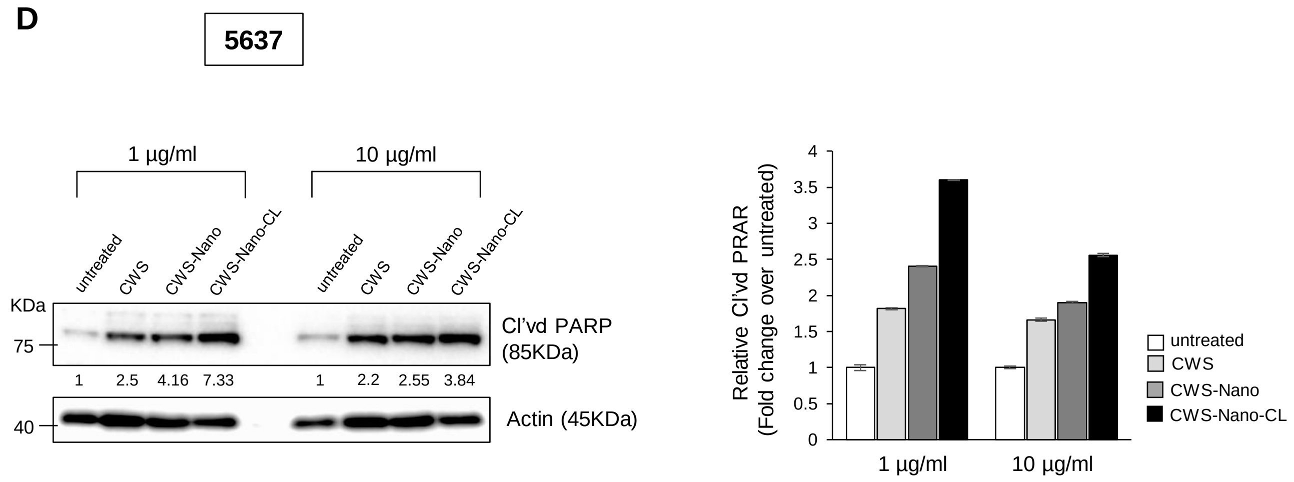

Figure S3. The quantitation results of Figure 2D.

Supplementary Fig. 4

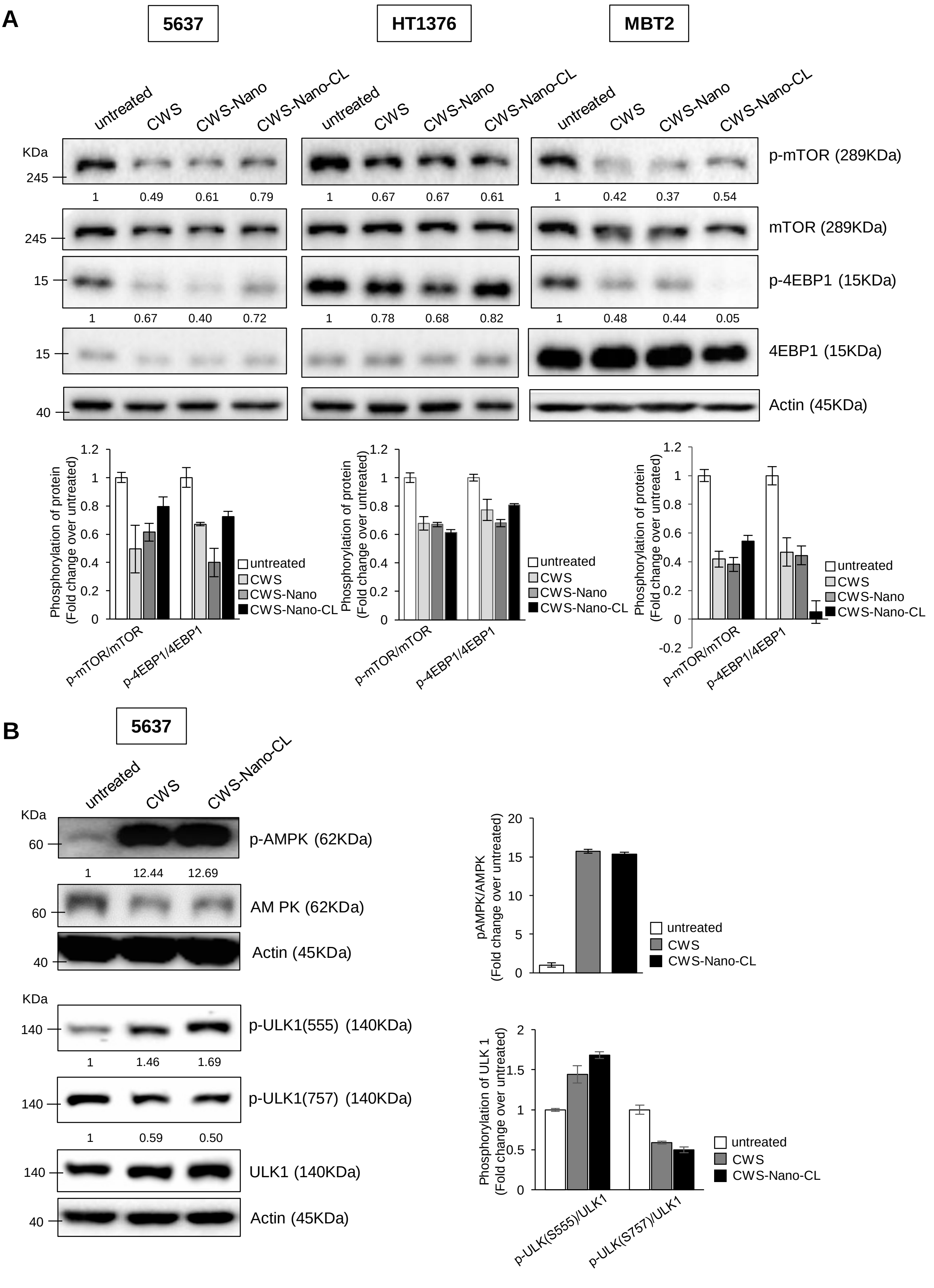

Figure S4. The quantitation results of Figure 3A and 3B.

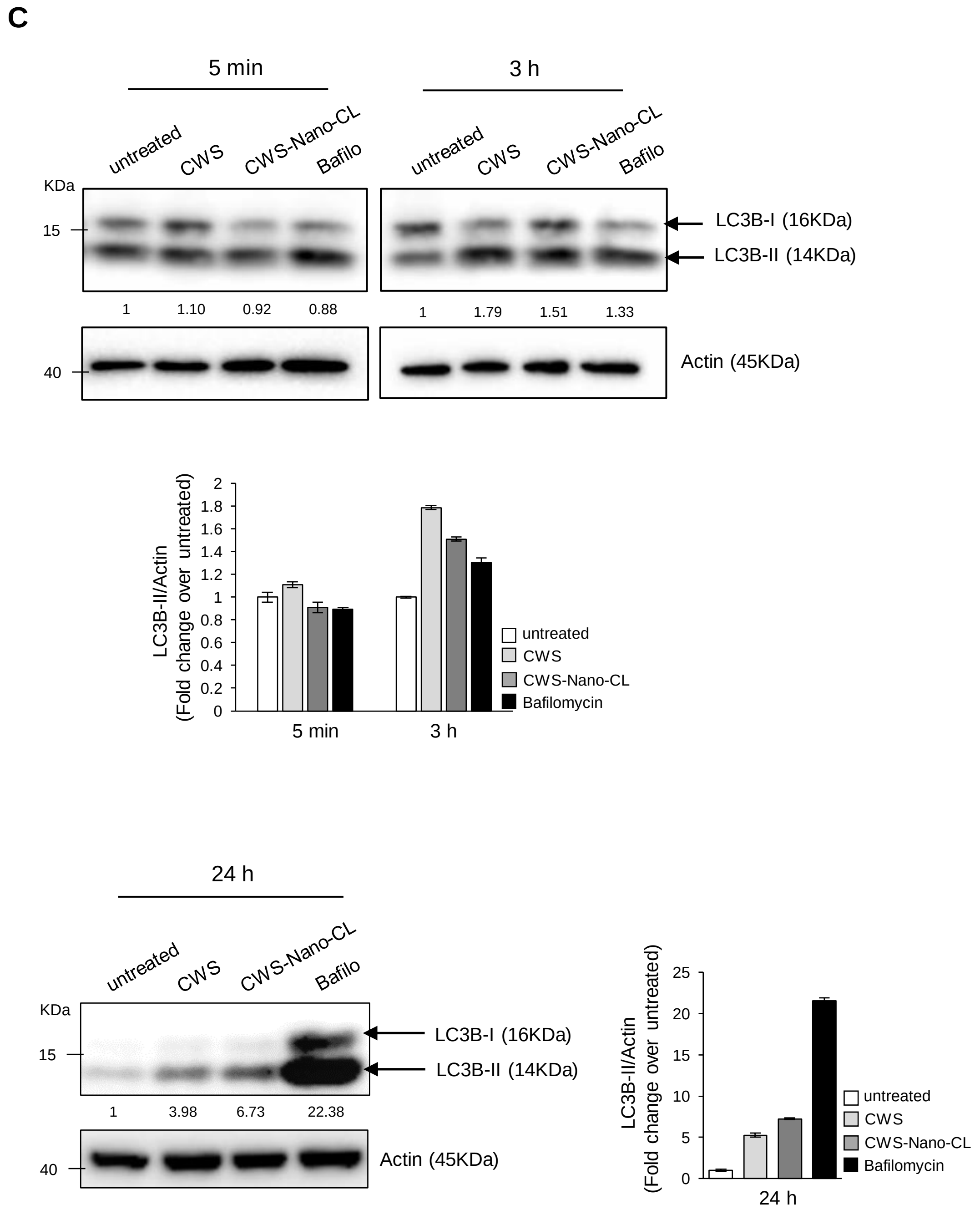

Figure S4. The quantitation results of Figure 3C

Supplementary Fig. 5

A

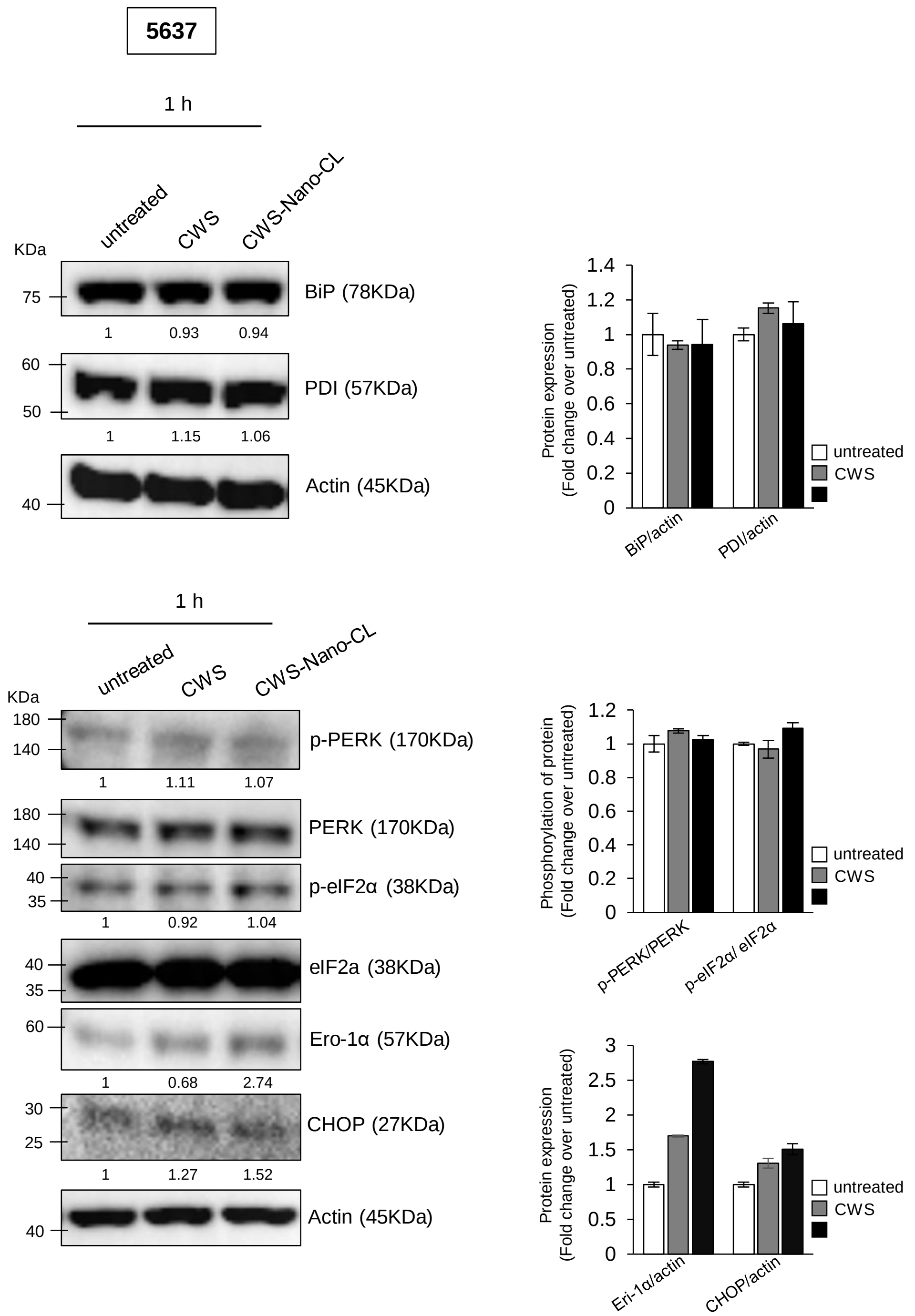

Figure S5. The quantitation results of Figure 5A

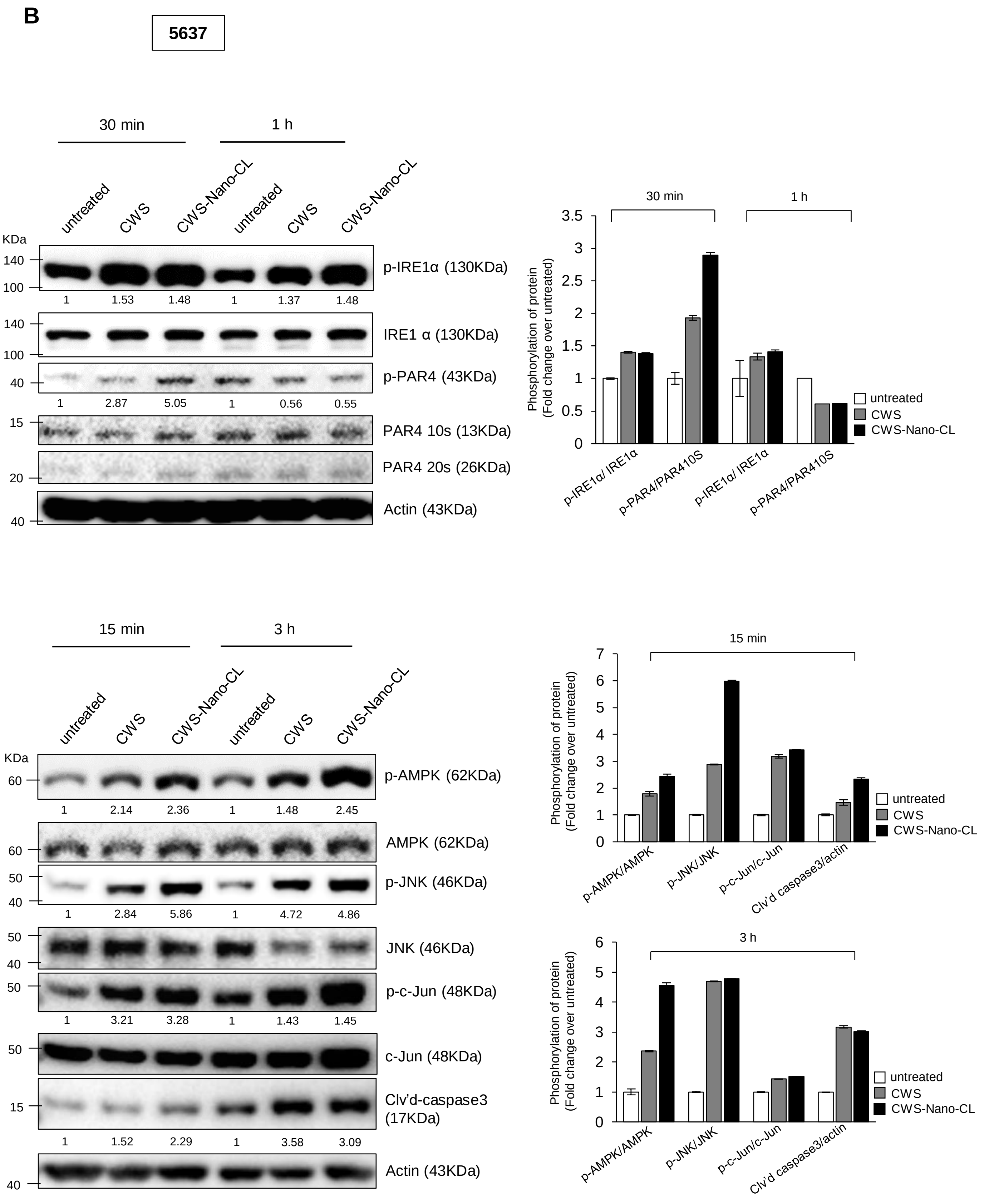

Figure S5. The quantitation results of Figure 5B

Supplementary Fig. 6

C

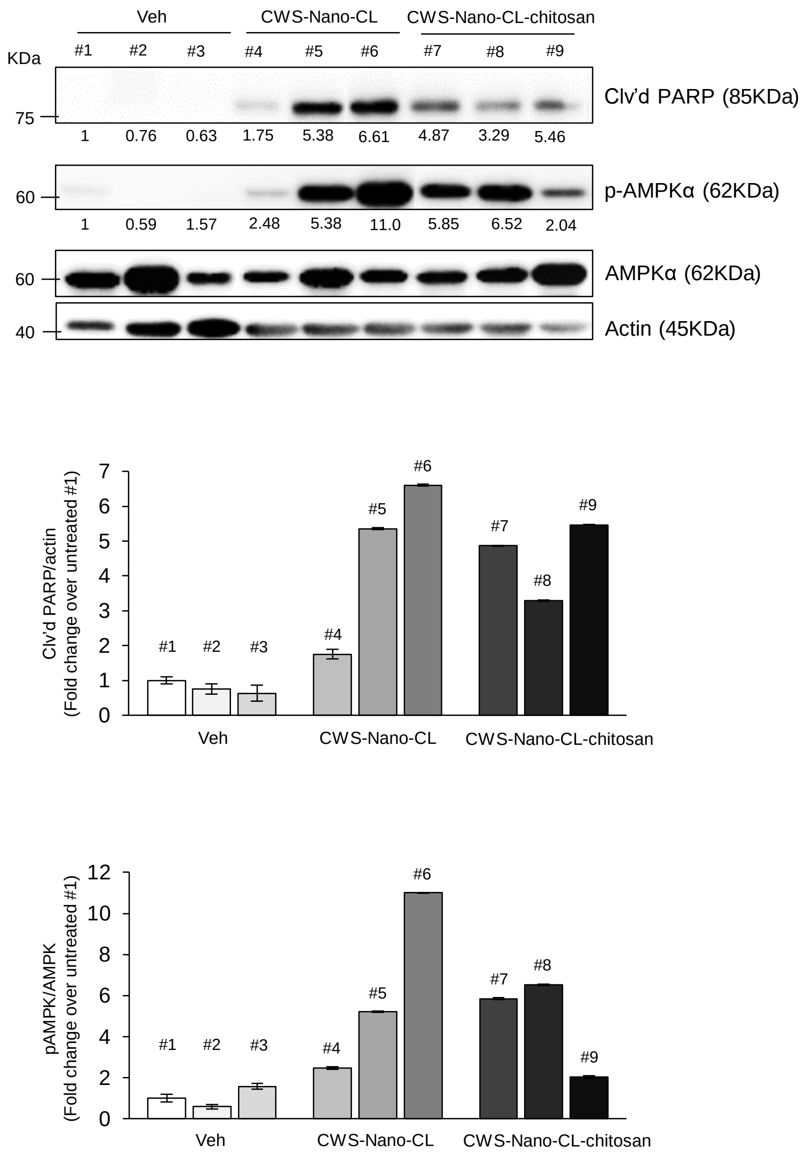

Figure S6. The quantitation results of Figure 6C

Figure S7: Whole blot showing all the bands with molecular weight markers on the Western blotting.

Figure 2D

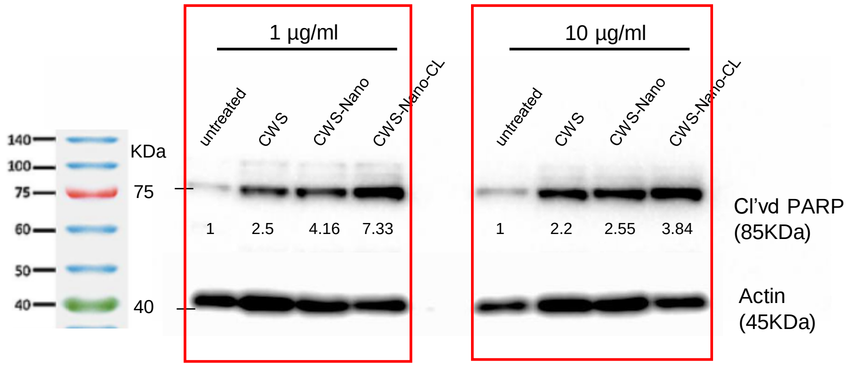

Figure 3A

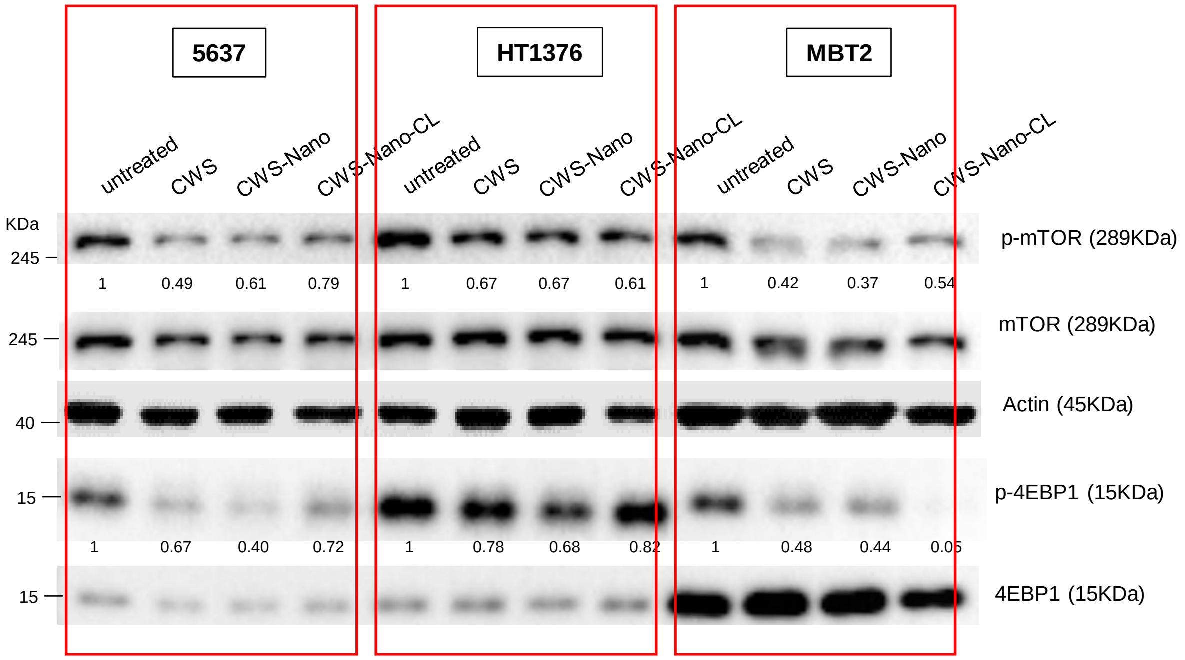

Figure 3B

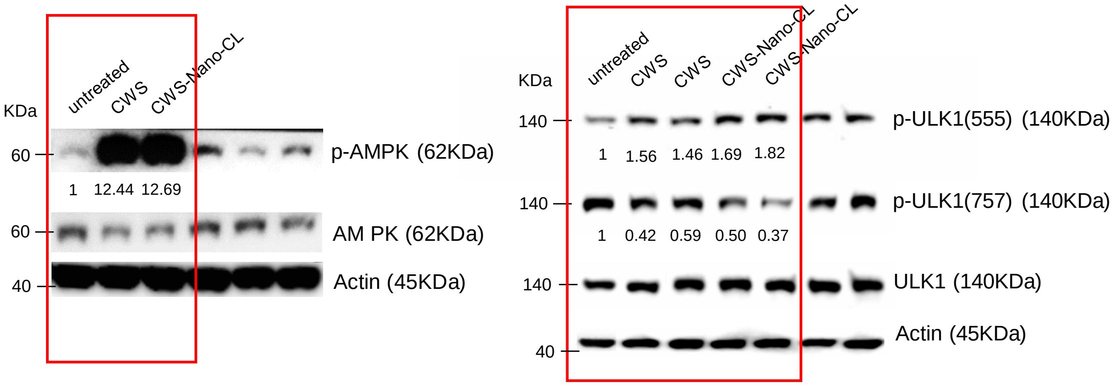

Figure 3C

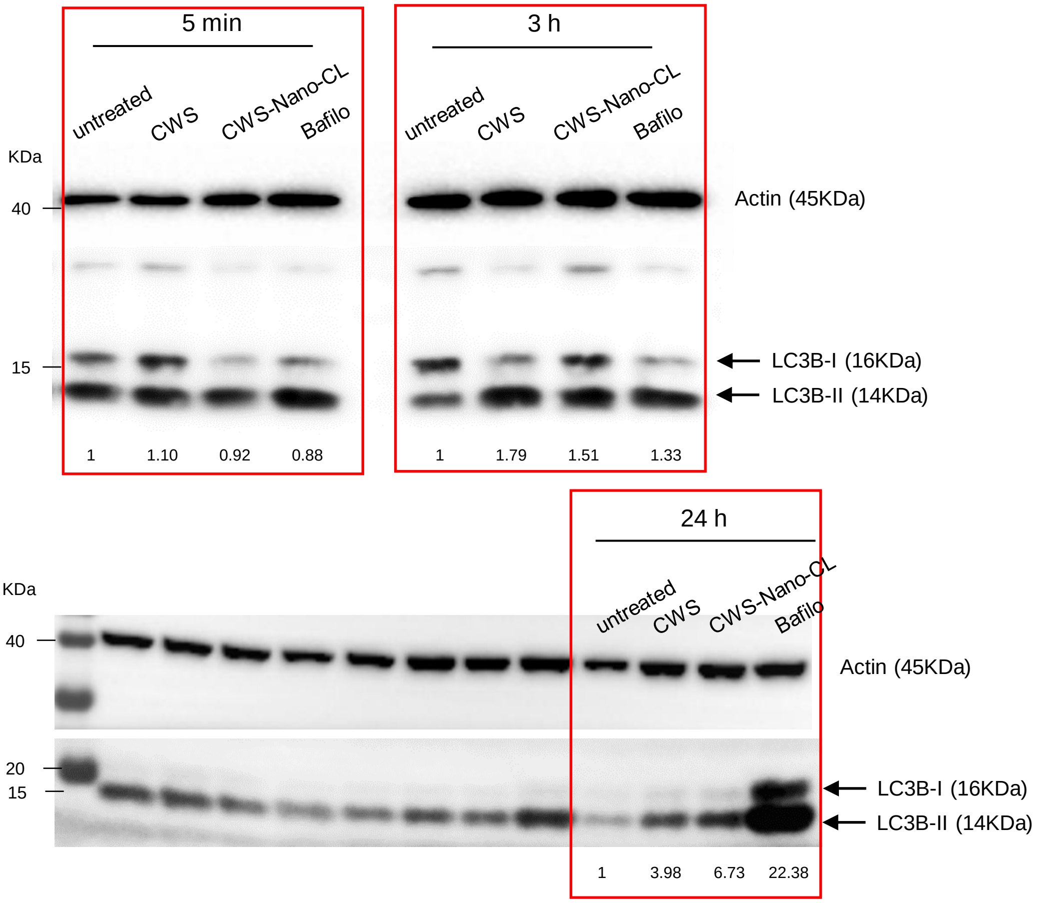

Figure 5A

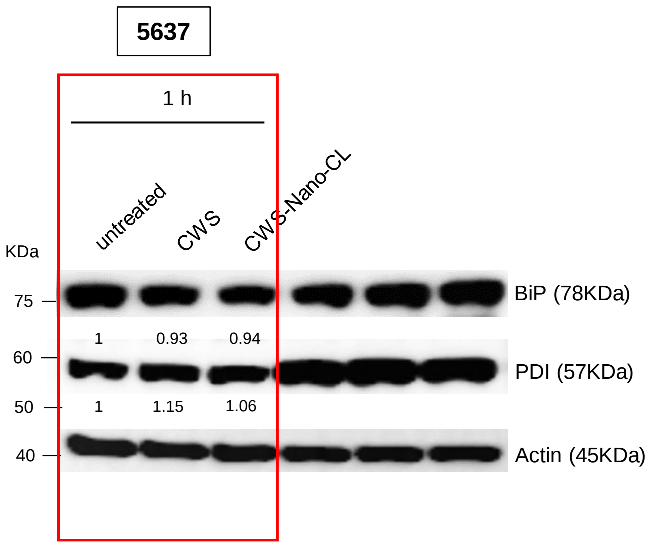

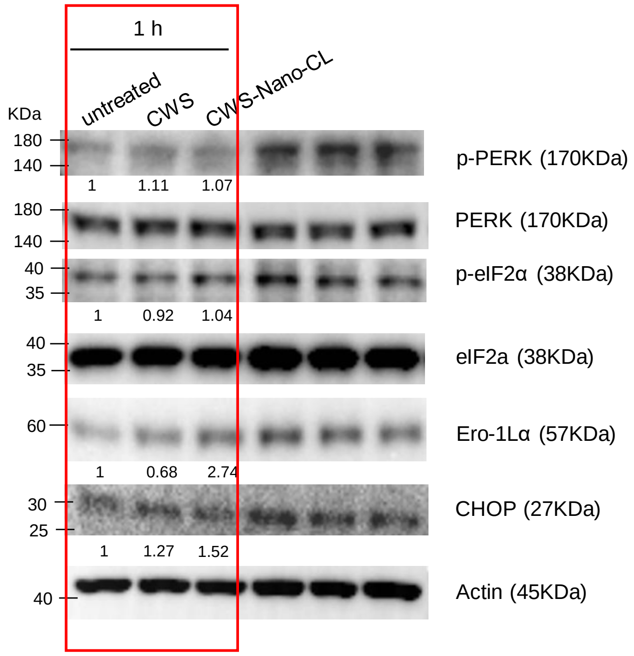

Figure 5B

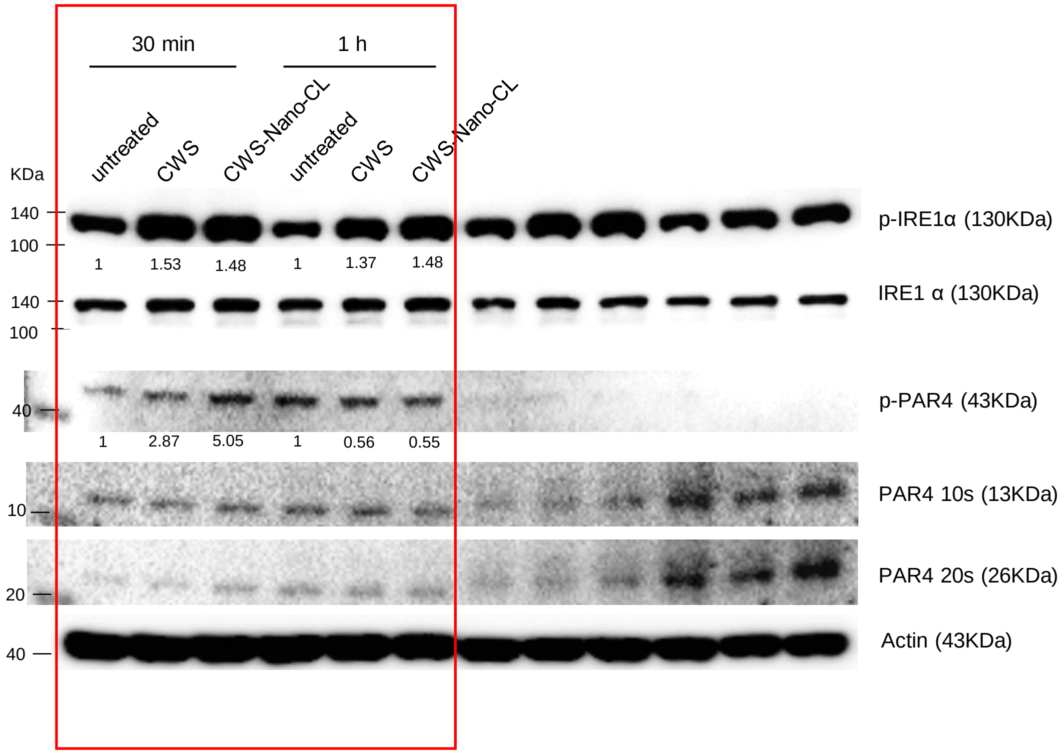

Figure 5B

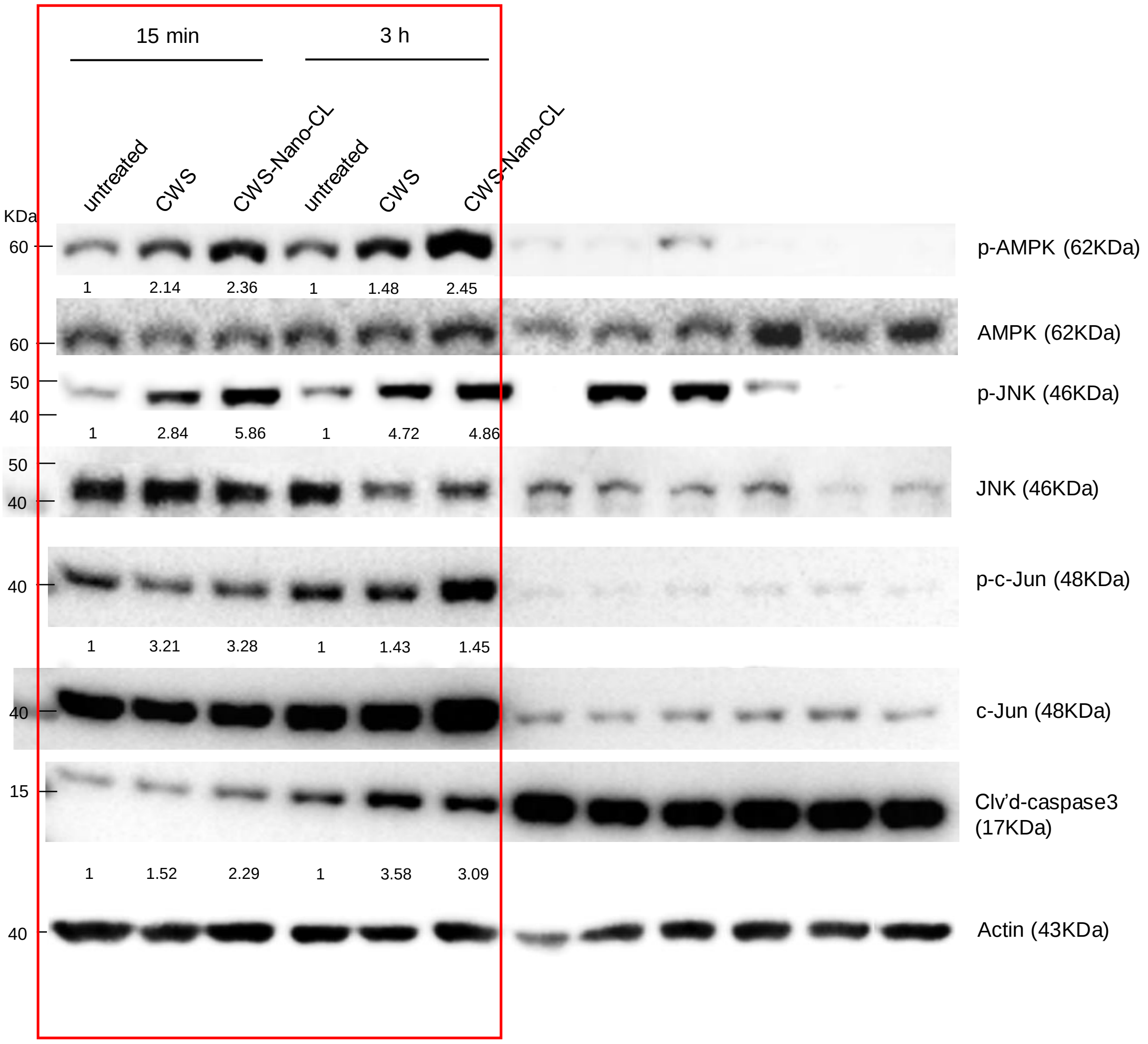

15 min
3 h
untreated
CWS
CWS-Nano-CL
untreated
CWS
CWS-Nano-CL
KDa
60
1
2.14
2.36
1
1.48
2.45
p-AMPK (62KDa)
60
AMPK (62KDa)
50
40
p-JNK (46KDa)
1
2.84
5.86
1
4.72
4.86
50
40
JNK (46KDa)
40
p-c-Jun (48KDa)
1
3.21
3.28
1
1.43
1.45
40
c-Jun (48KDa)
15
Clv'd-caspase3 (17KDa)
1
1.52
2.29
1
3.58
3.09
40
Actin (43KDa)

Figure 6C

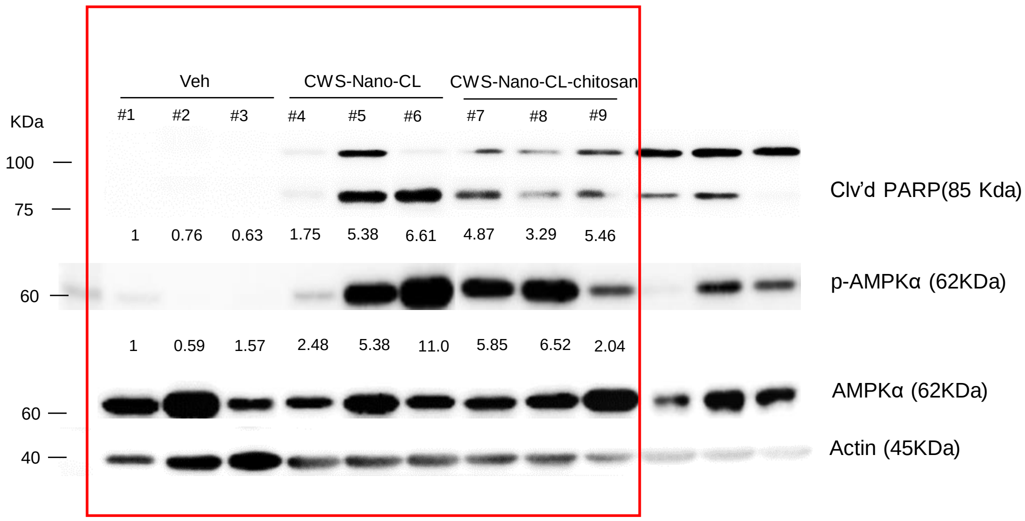

Figure S1

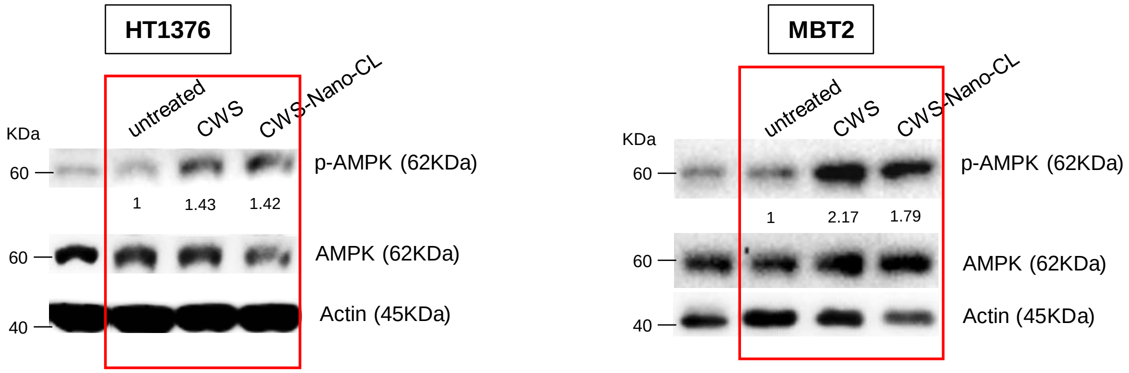

Supplement: Supplementary file 1 [file cancers-12-03679-s001.pdf]
